# Supplementary material for: EASTR: Identifying and eliminating systematic alignment errors in multi-exon genes
Source: Nat Commun. 2023 Nov 9;14:7223. doi: 10.1038/s41467-023-43017-4 (PMC10632439; doi:10.1038/s41467-023-43017-4)
Supplement: Supplementary file 1 — Supplementary Information [file 41467_2023_43017_MOESM1_ESM.pdf]

Supplementary Note 1: Validation of EASTR filtering within transcribed repetitive genomic regions

Identifying and eliminating erroneous splicing events within transcribed repetitive genomic regions, such as Human Endogenous Retroviruses (HERVs), poses unique challenges. The high sequence similarity among these elements creates a potential for misalignments. However, it is important to note that not all spliced alignments within these regions are erroneous, prompting concerns about potential over-filtering of valid splicing events by EASTR. To mitigate this, EASTR avoids indiscriminate removal of all junctions between repeat elements by examining alignments between upstream and downstream sequences around splice donor and splice acceptor sites. For retroviral elements that exploit splicing to diversify their compact genomes, it is uncommon for the upstream exon sequence to align precisely with the downstream intron sequence, and vice versa. Such alignment patterns are typically artifacts and do not represent genuine splice sites.

To assess EASTR's capability to discriminate valid junctions within repetitive regions we focused on the human genome segments containing HERVs, and we used SpliceAI[1] (version 1.3.1) to score all splice junctions overlapping HERV elements extracted from the alignments of the same dataset of 23 DLPFC human RNA-seq samples referenced in the main manuscript. Out of the 4,287 junctions detected by HISAT and the 2,344 identified by STAR, which showed overlap with HERV elements at either donor and acceptor sites, EASTR removed 261 and 205 junctions, respectively. In total there were 375 HERV-to-HERV junctions identified by either HISAT or STAR that were removed by EASTR and 804 HERV-to-HERV junctions identified by either aligner that were kept. If EASTR is effective in discriminating spurious junctions from genuine ones, the SpliceAI scores should be higher among the kept junctions. Indeed, the junctions removed by EASTR had lower average SpliceAI scores: 0.05 and 0.009 for acceptors and donors, respectively, while the retained junctions had significantly higher average scores: an acceptor score of 0.3 and a donor score of 0.17. In addition, none of the removed junctions had either splice donor or splice acceptor scores above 0.6, while among the set of retained junctions, there were 37 such instances (details provided in Supplementary Data 16 and17).

Supplementary Note 2: Questionable intron in *TCEANC* transcript

While the majority of MANE-selected isoforms serve as the preferred representatives for their corresponding genes, Sommer et al. identified several noteworthy exceptions [2] . Our analysis proposes that *TCEANC* may be another such exception. Exonization events are infrequent, and consecutive *Alu* element exonization requires a minimum of four specific mutations [3]. Moreover, *Alu* elements are susceptible to non-allelic homologous recombination (NAHR)[4] , which can produce deletions masquerading as introns during spliced alignments (Supplementary Figure 1C), necessitating meticulous evaluation of intron splicing events. The GTEx[5] RNA-seq data contain uniquely aligned spliced reads at this junction, possibly indicating a deletion. Although prevalent structural variant databases, such as dbVar[6] and gnomAD[7], report a chimeric *Alu*-producing duplication in this region (Supplementary Figure 1B), further investigation is required to validate this junction, as additional recombinations, including deletions, may remain undetected due to factors such as structural variant caller limitations with short reads and the abundance of *Alu* elements. Furthermore, we utilized SpliceAI[8] to

evaluate the acceptor and donor splice sites. The evaluation included the entire intron and an additional 200bp sequence upstream the donor and 200bp sequence downstream the acceptor. Following the guidelines from the SpliceAI manual (https://github.com/Illumina/SpliceAI), we included an extra 5,000bp on both the donor and acceptor side, resulting in a 10,000bp of flanking sequence context. Consequently, the full length of a splice site input into the SpliceAI model is 10,400bp plus the intron length. We calculated the average score from the five trained models for each site. The results, presented in Supplementary Figure 2, demonstrate that the second putative exonization event is notably weak.

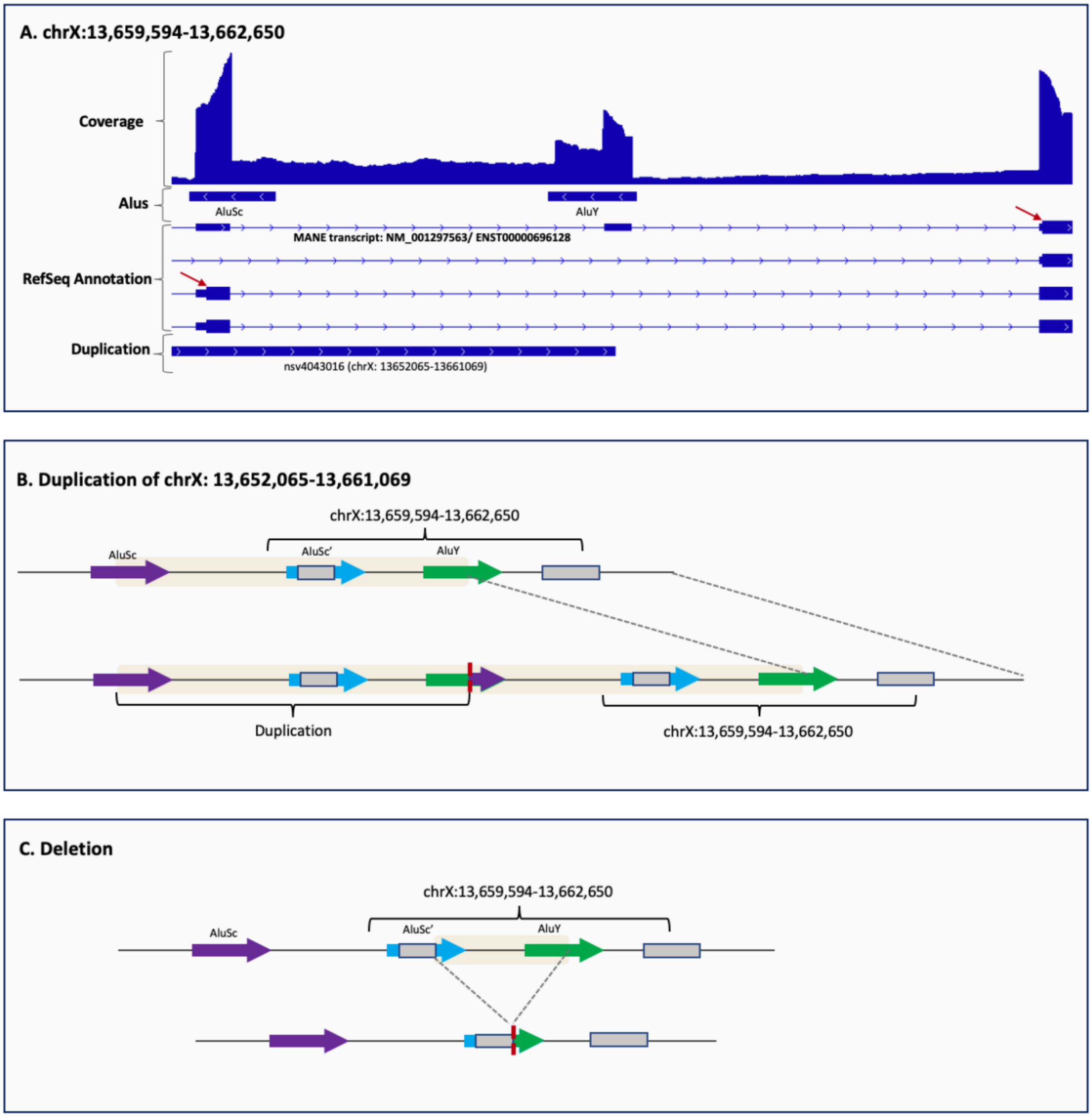

Supplementary Figure 1. Intronic Splicing Between Consecutive *Alu* Elements in *TCEANC* Gene. (A) The coverage track displays coverage from HISAT2 alignments across 9,795 GTEx samples. The *Alu*s track illustrates two consecutive *Alu* elements, with the RefSeq annotation below. The MANE transcript includes exons situated within the consecutive *Alu* elements. Red arrows mark the start of an ORF. This figure emphasizes our analysis of the MANE catalog, where we identified a *TCEANC* gene transcript, specifically NM_001297563 (CHESS: CHS.57562.1, GENCODE: ENST00000696128), harboring an intron warranting further investigation. GTEx RNA-seq data reveals uniquely aligned spliced reads at this junction, potentially indicating a deletion. Common structural variant databases, such as dbVar and gnomAD, report a chimeric *Alu*-producing duplication in this region, as shown in the duplication track. **(B)** The impact of the duplication shown in (A) is schematically represented, displaying the formation of a chimeric *Alu* between an *Alu*Y (green arrow) in the *TCEANC* gene and an upstream *Alu*Sc (purple arrow). The blue arrow depicts an *Alu*Sc' element within the *TCEANC* gene. Arrows depict *Alu* elements, the yellow rectangle highlights the region of duplication, and the breakpoint is indicated by a red dashed line. Gray rectangles represent exons. **(C)** Schematic representation of a hypothesized deletion resulting from NAHR.

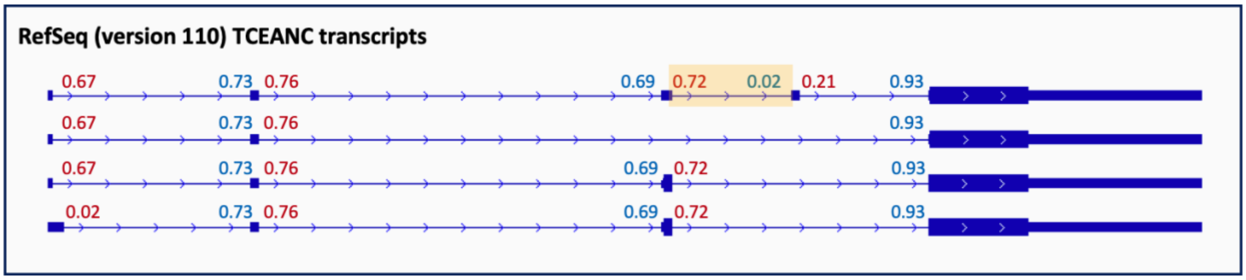

Supplementary Figure 2. SpliceAI donor and acceptor scores for introns in the *TCEANC* gene. The figure displays all *TCEANC* RefSeq transcripts and the SpliceAI scores for donor (in red text) and acceptor (in blue text) splice sites. The questionable intron is highlighted in yellow. The SpliceAI acceptor site score for the questionable intron is 0.02, more than 30 times lower than all other acceptor scores in this gene. Moreover, the exon following the questionable intron exhibits a low donor site score. The combination of the low acceptor score at the 5' end and the low donor score at the 3' end of the exon supports our hypothesis that this splice site is likely spurious.

Supplementary Note 3: Incomplete Annotation and Erroneous Transcripts in the *NPIPB3* Gene

The NPIP gene family, like the NBPF family examined in the results section, has expanded in primates through segmental duplications. The length of the VNTR region at the carboxy terminus is a defining characteristic that distinguishes paralogous copies[9]. We found that none of the protein coding transcripts in RefSeq, CHESS, or GENCODE gene catalogs fully cover the characteristic VNTR region at the carboxy terminus of *NPIPB3* (Supplementary Figure 3). Furthermore, this protein-coding gene is entirely absent from the MANE gene catalog. The sawtooth pattern observed in the coverage of spliced alignments suggests the presence of many erroneous spliced alignments in this repetitive region. Our findings are supported by the UCSC genome browser visualization of the CHM13 liftOver track, which reveals a nearby 252bp deletion in CHM13 relative to GRCh38. This deletion exactly matches the length of the intron in RefSeq transcript NM_130464 (CHESS3 transcript CHS.19306.24), strongly suggesting that it is a VNTR indel.

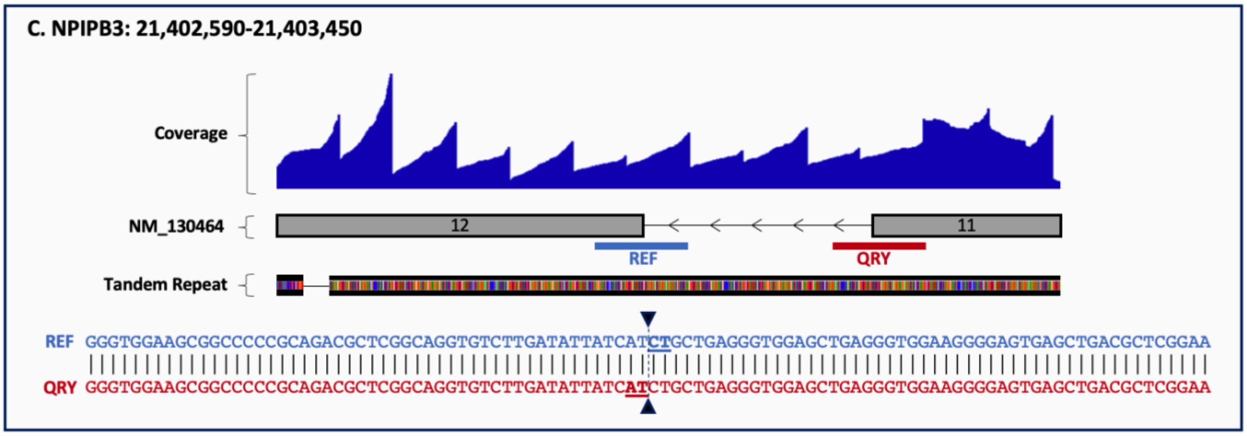

Supplementary Figure 3. Error in annotation of *NPIPB3* transcript NM_130464 caused by a VNTR polymorphism. A 252bp intron is incorrectly inserted between exons 11 and 12. The 100bp alignment presented below the transcripts displays identical upstream and downstream sequences flanking the intron in NM_130464, with the splice site indicated by inverted triangles. The erroneous intron's length is twice the size of the 126bp tandem repeat. The coverage track presents the coverage from 9,795 GTEx samples.

Supplementary Note 4: Errors in TAIR 10.1 gene annotation

Supplementary Figure 4 presents an example of erroneous splicing between putative tandem gene duplications, highlighting the need for careful evaluation and refinement of gene annotation methods.

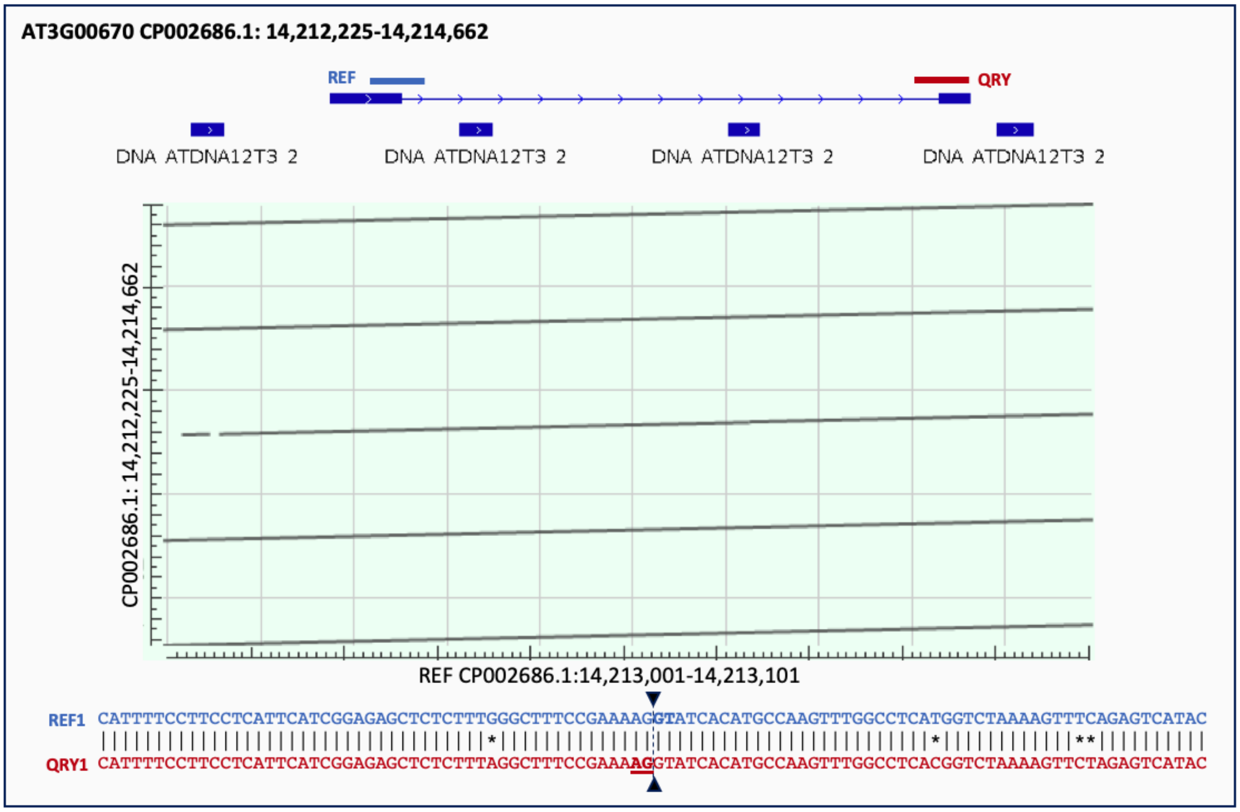

***Supplementary Figure 4*. Erroneous Splicing between tandemly duplicated regions in *A. thaliana*.** The top track displays the reference transcript for gene AT3G00670. The TE annotation track beneath the transcript illustrates tandemly duplicated transposons. The dot plot, generated using NCBI blastn[10] and located below the transcript and TE annotation tracks, features the reference query (REF) on the x-axis and the region presented in the transcript track on the y-axis. The reference sequence is observed to appear four times in this region. Alignments of the upstream and downstream intron-flanking sequences (REF and QRY) in transcripts exhibit 96% homology.

Supplementary Note 5: Effect of changing alignment parameters versus EASTR filtering

As discussed in the main text, alignment algorithms often show preferential treatment for spliced alignments over contiguous alignments. This can lead to inaccurate splice alignments, and consequently misassembled transcripts, particularly within repetitive regions of the genome.

To investigate if penalizing spliced alignments more heavily, relative to contiguous alignment, could enhance aligner performance, we built a HISAT index without reference annotation and executed two alignment runs:

- A **"no penalty"** run, where we kept HISAT2 default splice penalty of zero.
- A **"with penalty"** run where we applied the "pen-cansplice" flag to enforce a canonical splicing penalty of 12.

Supplementary Table 1 demonstrates that the "with penalty" approach consistently resulted in fewer matches to reference junctions across all samples, 6% lower than the number of

reference junctions that were kept by filtering with EASTR. While altering the penalty for splicing reduced novel splice junctions, it did so at the cost of transcript level precision and sensitivity (Supplementary **Table 2**). On the contrary, filtering the default HISAT alignments with EASTR, improved both measures of transcript reconstruction accuracy.

Supplementary Table 1: Comparative splice junction counts in 23 human samples. Comparative analysis of splice junction counts identified in 23 human samples using different approaches, including "no penalty", "with penalty", and "no penalty + EASTR" (where "no penalty" is followed by EASTR filtering).

	Reference Junctions			Novel Junctions		
**SRR_ID**	**No penalty**	**No Penalty + EASTR**	**With Penalty**	**No Penalty**	**No Penalty + EASTR**	**With Penalty**
SRR10689468	143,858	143,847	135,264	62,432	54,797	39,533
SRR10689469	116,735	116,731	105,598	23,700	20,547	13,610
SRR10689470	146,082	146,076	137,744	58,146	52,673	37,739
SRR10689471	110,882	110,878	99,558	26,118	20,552	13,869
SRR10689472	155,666	155,651	147,706	69,967	62,882	44,637
SRR10689473	172,416	172,404	164,170	117,960	100,748	66,689
SRR10689474	148,114	148,112	140,030	55,266	50,604	35,911
SRR10689475	154,706	154,697	146,152	79,466	68,325	45,225
SRR10689476	167,883	167,875	160,716	79,594	73,524	53,513
SRR10689477	132,629	132,625	123,558	33,129	30,527	21,360
SRR10689478	144,471	144,463	135,766	64,894	58,068	41,150
SRR10689479	160,223	160,215	153,101	70,839	65,750	48,091
SRR10689480	106,028	106,024	96,442	25,803	21,290	15,340
SRR10689481	155,315	155,306	148,417	77,060	70,475	51,445
SRR10689482	142,761	142,756	134,759	57,420	51,935	36,208
SRR10689483	174,557	174,546	167,953	145,498	133,955	97,269
SRR10689484	155,699	155,690	147,578	85,076	72,686	48,741
SRR10689485	161,961	161,951	154,960	75,499	69,087	50,080
SRR10689486	168,025	168,017	160,337	106,456	96,832	65,943
SRR10689487	193,639	193,620	187,594	227,806	209,864	149,091
SRR10689488	123,322	123,314	112,095	44,891	33,094	21,452
SRR10689489	166,168	166,155	157,234	108,374	95,957	59,709
SRR10689490	98,575	98,570	90,996	40,180	36,986	25,410

Supplementary Table 2: Transcript reconstruction precision and sensitivity metrics for different alignment approaches. Sensitivity and precision of transcript assembly in 23 human RNA-seq samples. Note that the low sensitivity numbers are mostly due to the fact that not all genes from the annotation are expressed.

	Transcript level Sensitivity (%)			Transcript level Precision (%)		
	No Penalty	No Penalty + EASTR	With Penalty	No Penalty	No Penalty + EASTR	With Penalty
SRR10689468	7.3	7.3	6.7	29.8	30.2	27.5
SRR10689469	3.8	3.8	3.1	22.9	23.0	18.5
SRR10689470	8.7	8.7	8.0	36.7	37.2	34.6
SRR10689471	2.5	2.5	1.9	14.1	14.4	10.9
SRR10689472	8.9	8.9	8.2	35.5	36.0	33.3
SRR10689473	10.4	10.4	9.6	36.3	37.4	35.2
SRR10689474	9.0	9.0	8.3	39.7	40.1	37.2
SRR10689475	7.9	7.9	7.1	32.9	33.6	30.1
SRR10689476	10.7	10.8	10.2	43.8	44.4	42.2
SRR10689477	6.0	6.0	5.3	34.1	34.3	29.9
SRR10689478	8.0	8.0	7.3	32.4	32.9	30.3
SRR10689479	10.3	10.3	9.6	44.2	44.7	42.4
SRR10689480	3.1	3.1	2.6	19.9	20.2	16.0
SRR10689481	9.5	9.6	9.0	41.2	41.8	39.4
SRR10689482	7.6	7.6	6.8	37.0	37.5	34.0
SRR10689483	11.8	11.8	11.3	40.4	41.1	40.3
SRR10689484	7.9	7.9	7.2	30.5	31.2	28.4
SRR10689485	9.9	9.9	9.2	41.5	42.0	39.5
SRR10689486	10.5	10.5	9.7	39.1	39.7	37.7
SRR10689487	14.1	14.1	13.7	39.9	40.7	40.7
SRR10689488	3.1	3.1	2.5	14.7	15.2	11.8
SRR10689489	9.6	9.6	8.7	36.9	37.7	34.8
SRR10689490	5.3	5.3	4.8	25.1	25.3	23.5

Supplementary Note 6: Analysis of shared spurious junctions across multiple samples

We analyzed 489 RNA-seq samples of heart tissues from GTEx in order to investigate whether spurious junctions detected by EASTR were shared across multiple samples or unique to individual ones. To this end, we extracted junctions from HISAT2 alignments of all samples and consolidated them into a single BED file. Only junctions supported by more than 5 alignments across the whole dataset were included. We then used EASTR to identify spurious junctions within this set.

As shown in Supplementary Figure 5 spurious junctions are often shared across samples, indicating a systematic pattern in these spurious alignments. This observation calls attention to the limitations of employing simple redundancy filtering techniques commonly used in studies, as it may not be adequate to remove shared alignment artifacts, and emphasizes the need for more refined methods to identify and mitigate such artifacts.

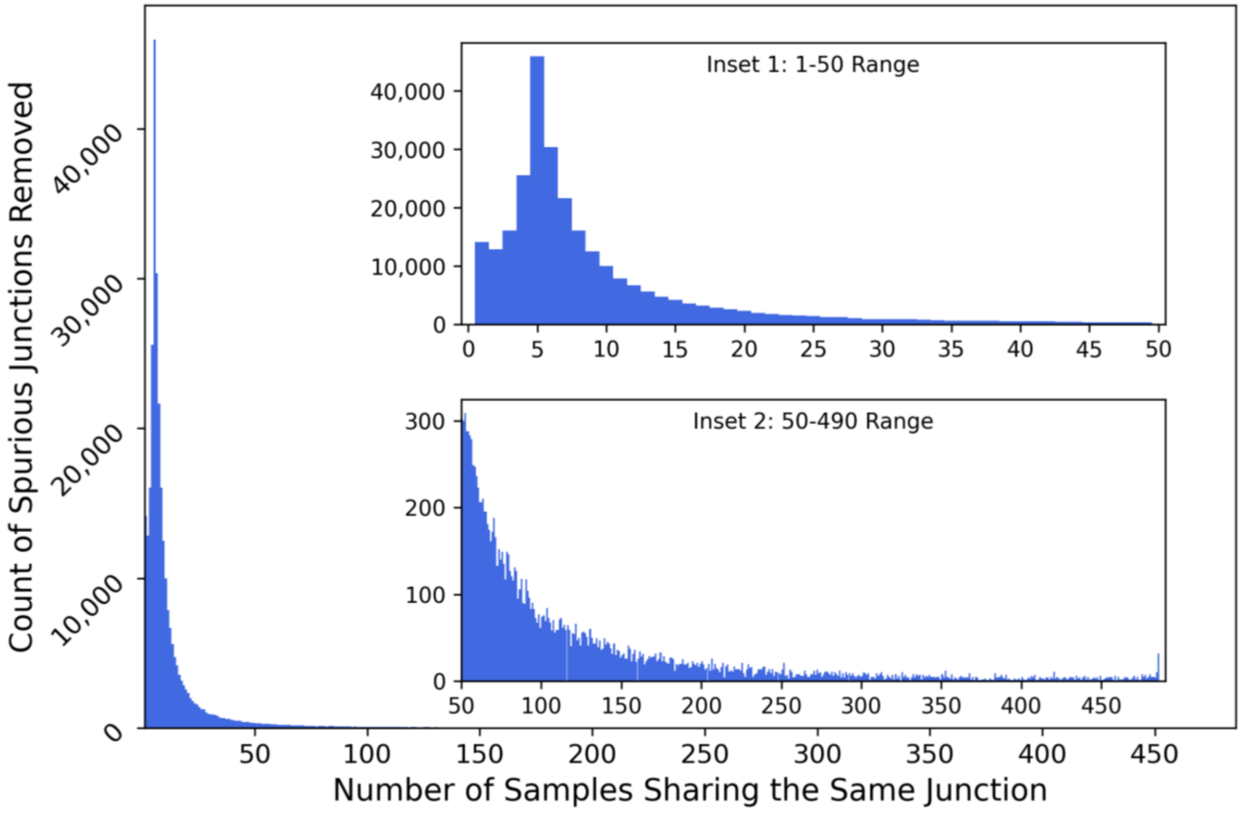

Supplementary Figure 5. Analysis of shared spurious junctions across multiple heart tissue samples from GTEx. The main plot illustrates the count of spurious junctions removed, categorizing them by the number of samples that share each junction. Two insets provide detailed views of specific ranges. Inset 1 (top right) zooms in on the 1-50 sample range, revealing the distribution of junctions that are shared by a smaller number of samples. Inset 2 (bottom right) focuses on the 50-490 sample range, illustrating the distribution of junctions shared by a larger number of samples.

Supplementary Note 7: EASTR Runtime and memory requirements

EASTR is designed to accommodate various input formats, enhancing flexibility and adaptability for different data types and requirements. The following options (with their associated outputs) are available:

- **Annotation GTF File:** EASTR can take an annotation GTF file as input and output a BED file containing the identified spurious junctions.
- **Individual BAM File:** If provided with a BAM file, EASTR can generate both a BED file of spurious junctions and a filtered BAM file with the spurious junctions removed.
- **List of BAM Files:** EASTR also accepts a list of BAM files, producing for each file a BED file of removed junctions and a corresponding filtered BAM file. This option is recommended to enhance efficiency by avoiding redundant checks of shared junctions among samples.

The runtime and memory requirements for the input/output types listed above are as follows:

- **Human RefSeq Annotation:** Running EASTR on the human RefSeq annotation required approximately 1 minute and 32 seconds of wall clock time, utilizing a maximum resident set size (RSS) of 3.2 GB.
- **Individual BAM Files:** Processing 23 HISAT2-aligned human data BAM files with filtered output resulted in a runtime ranging from 3.2 to 46.8 CPU minutes per file, with a consistent maximum RSS of 3.2 GB.
- **Batch Processing of BAM Files:** When executing EASTR on a batch of 46 BAM files (23 human data files aligned with HISAT and STAR) using 12 CPUs, the wall clock time was approximately 53 minutes and 17 seconds. The cumulative user time reached about 393.6 CPU minutes, with a maximum RSS of 4.3 GB.

Supplementary Note 8: EASTR Parameter selection
EASTR employs alignment parameters that can be adjusted, particularly the overhang length (default value = 50bp) and match score (default value = 3), to detect spurious junctions. To explore the impact of varying these two parameters, we looked at the sensitivity of detecting spurious splice junctions that overlap with *Alu* elements oriented in the same direction, as *Alu* elements have diverged over time and splicing between consecutive *Alu* elements is considered rare.

Our analysis of *Alu*-to-*Alu* splice junctions extracted from 23 DLPFC human RNA-seq samples revealed that increasing both the match score and the overhang improves detection sensitivity, likely by allowing EASTR to detect elements with higher divergence rates (Supplementary Table 3). Note increasing the overhang to 150bp when running EASTR on the 23 human samples did not change StringTie's transcript-level sensitivity or precision but did have a significant impact on runtime and memory requirements.

Supplementary Table 3: Sensitivity Analysis of Different EASTR Parameters. Sensitivity of detecting *Alu*-to-*Alu* spurious splicing with EASTR by using various combinations of match score and overhang length.

Match Score	Overhang (bp)	Sensitivity
3	50	84.3
3	100	89.6
3	150	93.3
2	50	58.2
2	100	76.5
2	150	83.0

Supplementary References

1. Jaganathan, K. *et al*. Predicting Splicing from Primary Sequence with Deep Learning. *Cell* **176**, 535-548.e24 (2019).

2. Sommer, M. J. *et al*. Structure-guided isoform identification for the human transcriptome. *eLife* **11** (2022).

3. Gal-Mark, N., Schwartz, S. & Ast, G. Alternative splicing of Alu exons--two arms are better than one. *Nucleic Acids Research* **36**, 2012-2023 (2008).

4. Balachandran, P. *et al*. Transposable element-mediated rearrangements are prevalent in human genomes. *Nature communications* **13**, 7115 (2022).

5. Carithers, L. J. & Moore, H. M. The Genotype-Tissue Expression (GTEx) Project. *Biopreservation and biobanking* **13**, 307-308 (2015).

6. Lappalainen, I. *et al*. dbVar and DGVa: public archives for genomic structural variation. *Nucleic Acids Research* **41**, D936-D941 (2013).

7. Collins, R. L. *et al*. A structural variation reference for medical and population genetics. *Nature (London)* **581**, 444-451 (2020).

8. Jaganathan, K. *et al*. Predicting Splicing from Primary Sequence with Deep Learning. *Cell* **176**, 535-548.e24 (2019).

9. Cantsilieris, S. *et al*. An evolutionary driver of interspersed segmental duplications in primates. *Genome Biology* **21**, 202 (2020).

10. Altschul, S. F., Gish, W., Miller, W., Myers, E. W. & Lipman, D. J. Basic local alignment search tool. *J. Mol. Biol.* **215**, 403-410 (1990).
