# Supplementary material for: EASTR: Identifying and eliminating systematic alignment errors in multi-exon genes
Source: Nat Commun. 2023 Nov 9;14:7223. doi: 10.1038/s41467-023-43017-4 (PMC10632439; doi:10.1038/s41467-023-43017-4)
Supplement: Supplementary file 3 — Description of Additional Supplementary Files [file 41467_2023_43017_MOESM3_ESM.pdf]

Description of Additional Supplementary Files

File Name: Supplementary Data 1
Description: Junctions and Alignments data for Human DLPFC samples.

File Name: Supplementary Data 2
Description: Junctions and Alignments data for A. thaliana samples.

File Name: Supplementary Data 3
Description: Junctions and Alignments data for Zea mays samples.

File Name: Supplementary Data 4
Description: GffCompare Counts for Human DLPFC samples.

File Name: Supplementary Data 5
Description: GffCompare Counts for A. thaliana samples.

File Name: Supplementary Data 6
Description: GffCompare Counts for Zea mays samples.

File Name: Supplementary Data 7
Description: GffCompare Precision and Sensitivity metrics for Human DLPFC samples.

File Name: Supplementary Data 8
Description: GffCompare Precision and Sensitivity metrics for A. thaliana samples.

File Name: Supplementary Data 9
Description: GffCompare Precision and Sensitivity metrics for Zea mays samples.

File Name: Supplementary Data 10
Description: EASTR flagged introns in Human RefSeq v110 Annotation.

File Name: Supplementary Data 11
Description: EASTR flagged introns in Human GENCODE v41 Annotation.

File Name: Supplementary Data 12
Description: EASTR flagged introns in Human CHESS v3.0 Annotation.

File Name: Supplementary Data 13
Description: EASTR flagged introns in Human MANE v1.0 Annotation.

File Name: Supplementary Data 14
Description: EASTR flagged introns in Zea Mays B73 NAM 5.0 Zm00001eb.1 Annotation.

File Name: Supplementary Data 15
Description: EASTR flagged introns in Arabidopsis Thaliana TAIR10.1 Annotation.

File Name: Supplementary Data 16
Description: SpliceAI scores for EASTR flagged HERV-to-HERV junctions.

File Name: Supplementary Data 17
Description: SpliceAI scores for kept HERV-to-HERV junctions.
