# Supplementary material for: EASTR: Identifying and eliminating systematic alignment errors in multi-exon genes
Source: Nat Commun. 2023 Nov 9;14:7223. doi: 10.1038/s41467-023-43017-4 (PMC10632439; doi:10.1038/s41467-023-43017-4)
Supplement: Supplementary file 5 — Reporting Summary [file 41467_2023_43017_MOESM5_ESM.pdf]

nature portfolio

Corresponding author(s): Ida Shinder, Mihaela Pertea

Last updated by author(s): October 12, 2023

Reporting Summary

Nature Portfolio wishes to improve the reproducibility of the work that we publish. This form provides structure for consistency and transparency in reporting. For further information on Nature Portfolio policies, see our Editorial Policies and the Editorial Policy Checklist.

Statistics

For all statistical analyses, confirm that the following items are present in the figure legend, table legend, main text, or Methods section.

n/a	Confirmed	
☒	☐	The exact sample size (n) for each experimental group/condition, given as a discrete number and unit of measurement
☒	☐	A statement on whether measurements were taken from distinct samples or whether the same sample was measured repeatedly
☒	☐	The statistical test(s) used AND whether they are one- or two-sided<br>*Only common tests should be described solely by name; describe more complex techniques in the Methods section.*
☒	☐	A description of all covariates tested
☒	☐	A description of any assumptions or corrections, such as tests of normality and adjustment for multiple comparisons
☒	☐	A full description of the statistical parameters including central tendency (e.g. means) or other basic estimates (e.g. regression coefficient) AND variation (e.g. standard deviation) or associated estimates of uncertainty (e.g. confidence intervals)
☒	☐	For null hypothesis testing, the test statistic (e.g. F, t, r) with confidence intervals, effect sizes, degrees of freedom and P value noted<br>*Give P values as exact values whenever suitable.*
☒	☐	For Bayesian analysis, information on the choice of priors and Markov chain Monte Carlo settings
☒	☐	For hierarchical and complex designs, identification of the appropriate level for tests and full reporting of outcomes
☒	☐	Estimates of effect sizes (e.g. Cohen's d, Pearson's r), indicating how they were calculated

Our web collection on statistics for biologists contains articles on many of the points above.

Software and code

Policy information about availability of computer code

Data collection	Raw sequencing (fastq) files were downloaded using the prefetch (v3.0.2) command from SRA-Toolkit.
Data analysis	EASTR 0.1.0 (https://github.com/ishinder/EASTR or DOI: 10.5281/zenodo.530774518)<br>HISAT2 2.2.1<br>STAR 2.7.8a_2021-03-08<br>StringTie2 2.2.2<br>samtools 1.13<br>bowtie2 2.4.4<br>gffcompare 0.12.6

For manuscripts utilizing custom algorithms or software that are central to the research but not yet described in published literature, software must be made available to editors and reviewers. We strongly encourage code deposition in a community repository (e.g. GitHub). See the Nature Portfolio guidelines for submitting code & software for further information.

Data

Policy information about availability of data

All manuscripts must include a data availability statement. This statement should provide the following information, where applicable:

- Accession codes, unique identifiers, or web links for publicly available datasets
- A description of any restrictions on data availability
- For clinical datasets or third party data, please ensure that the statement adheres to our policy

Human DLPFC dataset is available under accession PRJNA595606 [https://www.ncbi.nlm.nih.gov/bioproject/PRJNA595606].

Maize leaf dataset is available under accessions SRR10095075 [https://www.ncbi.nlm.nih.gov/sra/?term=SRR10095075], SRR10095076 [https://www.ncbi.nlm.nih.gov/sra/?term=SRR10095076], SRR10095077 [https://www.ncbi.nlm.nih.gov/sra/?term=SRR10095077].

The maize pollen dataset is available under accession numbers SRR3091548 [https://www.ncbi.nlm.nih.gov/sra/?term=SRR3091548], SRR3091717 [https://www.ncbi.nlm.nih.gov/sra/?term=SRR3091717], SRR3094513 [https://www.ncbi.nlm.nih.gov/sra/?term=SRR3094513]

A. thaliana dataset is available under accessions SRR14056780 [https://www.ncbi.nlm.nih.gov/sra/?term=SRR14056780], SRR14056781 [https://www.ncbi.nlm.nih.gov/sra/?term=SRR14056781], SRR14056782 [https://www.ncbi.nlm.nih.gov/sra/?term=SRR14056781], SRR16596898 [https://www.ncbi.nlm.nih.gov/sra/?term=SRR16596898], SRR16596890 [https://www.ncbi.nlm.nih.gov/sra/?term=SRR16596890], SRR16596900 [https://www.ncbi.nlm.nih.gov/sra/?term=SRR16596900]

GRCh38 genome assembly as available under RefSeq accession GCF_000001405.39 [https://www.ncbi.nlm.nih.gov/assembly/GCF_000001405.39/])
TAIR10.1 genome assembly is available under RefSeq accession GCF_000001735.4 [https://www.ncbi.nlm.nih.gov/assembly/GCF_000001735.4/])
B73 NAM 5.0 genome assembly is available under RefSeq accession GCF_902167145.1 [https://www.ncbi.nlm.nih.gov/assembly/GCF_902167145.1/])

Transcriptome assemblies generated in this study are available for download from the following FTP link: ftp://ftp.ccb.jhu.edu/pub/EASTR. There are no restrictions on data use or controlled access conditions.

Research involving human participants, their data, or biological material

Policy information about studies with human participants or human data. See also policy information about sex, gender (identity/presentation), and sexual orientation and race, ethnicity and racism.

Reporting on sex and gender	N/A
Reporting on race, ethnicity, or other socially relevant groupings	N/A
Population characteristics	N/A
Recruitment	N/A
Ethics oversight	N/A

Note that full information on the approval of the study protocol must also be provided in the manuscript.

Field-specific reporting

Please select the one below that is the best fit for your research. If you are not sure, read the appropriate sections before making your selection.

☒ Life sciences ☐ Behavioural & social sciences ☐ Ecological, evolutionary & environmental sciences

For a reference copy of the document with all sections, see nature.com/documents/nr-reporting-summary-flat.pdf

Life sciences study design

All studies must disclose on these points even when the disclosure is negative.

Sample size	Not applicable - the study does not involve a statistical analysis.
Data exclusions	No data were excluded from the analyses.
Replication	Not applicable. The results have been generated using open source software and the scripts to replicate the findings are also available.
Randomization	Our study focuses on computational analysis to improve alignment/assembly in publicly available datasets. We compare results for each

Randomization	sample before and after EASTR filtering, thus randomization is not applicable to our experimental design.
Blinding	Our study involves the computational analysis of publicly available datasets. The analyses are automated and do not involve subjective interpretations that could be influenced by prior knowledge or assumptions. Therefore, the concept of blinding is not applicable.

Reporting for specific materials, systems and methods

We require information from authors about some types of materials, experimental systems and methods used in many studies. Here, indicate whether each material, system or method listed is relevant to your study. If you are not sure if a list item applies to your research, read the appropriate section before selecting a response.

Materials & experimental systems

n/a	Involved in the study
☒	☐ Antibodies
☒	☐ Eukaryotic cell lines
☒	☐ Palaeontology and archaeology
☒	☐ Animals and other organisms
☒	☐ Clinical data
☒	☐ Dual use research of concern
☒	☐ Plants

Methods

n/a	Involved in the study
☒	☐ ChIP-seq
☒	☐ Flow cytometry
☒	☐ MRI-based neuroimaging
